# Summary of Joint European Hematology Association (EHA) and EuroBloodNet Recommendations on Diagnosis and Treatment of Methemoglobinemia

**DOI:** 10.1097/HS9.0000000000000660

**Published:** 2021-11-17

**Authors:** Achille Iolascon, Immacolata Andolfo, Roberta Russo, Wilma Barcellini, Elisa Fermo, Gergely Toldi, Stefano Ghirardello, David Rees, Richard Van Wijk, Antonis Kattamis, Patrick G. Gallagher, Noemi Roy, Ali Taher, Razan Mohty, Andreas Kulozik, Lucia De Franceschi, Antonella Gambale, Mariane De Montalembert, Gian Luca Forni, Cornelis L. Harteveld, Josef Prchal, Paola Bianchi, SWG of Red Cell and Iron of EHA and EuroBloodNet

**Affiliations:** ^1^ Dipartimento di Medicina Molecolare e Biotecnologie Mediche Università degli Studi di Napoli Federico II Napoli Italy; ^2^ CEINGE Biotecnologie Avanzate Napoli Italy; ^3^ UOS Fisiopatologia delle Anemie UO Ematologia Fondazione IRCCS Ca’ Granda Ospedale Maggiore Policlinico Milano Italy; ^4^ Department of Neonatology Birmingham Women's and Children's Hospital Birmingham United Kingdom; ^5^ Neonatal Intensive Care Unit Fondazione IRCCS Policlinico San Matteo Pavia Italy; ^6^ King's College Hospital King's College London United Kingdom; ^7^ Central Diagnostic Laboratory University Medical Center Utrecht Utrecht University Utrecht the Netherlands; ^8^ First Department of Pediatrics University of Athens Greece; ^9^ Departments of Pediatrics Pathology, and Genetics Yale University New Haven Connecticut USA; ^10^ Department of Haematology Oxford University Hospitals NHS Foundation Trust Oxford United Kingdom; ^11^ NIHR BRC Blood Theme Oxford United Kingdom; ^12^ Department of Haematology Oxford United Kingdom; ^13^ Division of Hematology and Oncology Department of Internal Medicine American University of Beirut Medical Center Beirut Lebanon; ^14^ Department of Pediatric Oncology Hematology and Immunology University of Heidelberg Hopp‐Children's Cancer Research Center (KiTZ) Heidelberg Germany; ^15^ Department of Medicine University of Verona and Azienda Ospedaliera Universitaria Verona Policlinico GB Rossi Verona Italy; ^16^ Department of Laboratory Medicine (DAIMedLab) UOC Medical Genetics ‘Federico II’ University Hospital Naples Italy; ^17^ Pediatrics Department Necker Hospital Paris France; ^18^ Centro della Microcitemia e Anemie Congenite Ospedale Galliera Genova Italy; ^19^ Department of Clinical Genetics/LDGA Leiden University Medical Center Leiden the Netherlands; ^20^ Hematology University of Utah & Huntsman Cancer Center Salt Lake City Utah USA

Methemoglobinemia is a rare disorder associated with oxidization of divalent ferrous iron of hemoglobin (Hb) to ferric iron of methemoglobin (MetHb), resulting from either inherited or acquired processes (Fig. 1). Acquired forms are the most common, mainly due to the exposure to substances that cause oxidation of the Hb both directly or indirectly. Inherited forms are due either to autosomal recessive variants in the *CYB5R3* gene (NADH diaphorase deficiency) or to autosomal dominant variants in the globin genes, collectively known as HbM disease^1^, ^2^ (Fig. 1 and Table 1).

**Table 1:** Forms and Symptoms of Methemoglobinemia

Disease	Transmission	Gene(s)	Symptoms
Drug exposure	Acquired	—	Cyanosis
Methemoglobinemia, type I	Autosomal recessive	*CYB5R3*	Cyanosis since birth
Methemoglobinemia, type II	Autosomal recessive	*CYB5R3*	Cyanosis since birth, Neurological involvement
Methemoglobinemia, type IV	Autosomal recessive	*CYB5A*	Cyanosis, 46,XY DSD
			Ambiguous genitalia
HbM disease	Autosomal dominant	*HBA1, HBA2, HBB, HBG1, HBG2*	Cyanosis since birth or after HbF/A switching, anemia
Unstable Hb	Autosomal dominant	*HBA1, HBA2, HBB, HBG1, HBG2*	Cyanosis, anemia

DSD = disorder of sexual differentiation.

Based on the severity of the enzyme deficiency and on *CYB5R3* genotype, NADH diaphorase deficiency can be classified into 2 different subtypes; type I, mainly due to missense variants that cause a production of an unstable enzyme purely in the red blood cells, is associated with MetHb levels above 25%, cyanosis, headache, fatigue, and dyspnea; of note, in these cases, cyanosis may be the only symptom since most of type I patients are asymptomatic; type II, caused by variants that lead to either low expression or low activity of the enzyme in all the tissues, is associated with alterations in the lipid metabolism and neurological involvement^3^ (Table 1).

**Figure 1:**
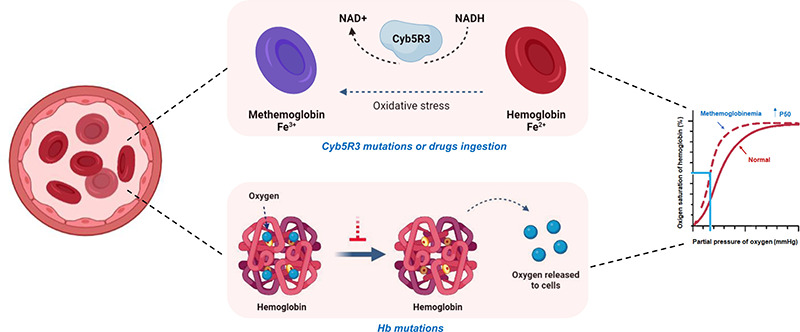
**Causes and effects of methemoglobinemia.** Schematic representation of mechanisms that cause methemoglobinemia. Methemoglobinemia can result from either inherited or acquired processes. The panel on the top of the figure is the representation of one of the hereditary forms of methemoglobinemia caused by mutations in *CYB5R3* gene encoding for the NADH cytochrome b5 reductase and of the acquired forms caused by drug ingestion or toxic exposure that account for the acceleration of Hb oxidization from the ferrous to the ferric state. The panel on the bottom shows the alterations of the hemoglobin caused by: mutations in the genes encoding alpha‐globin (*HBA1* and *HBA2*), beta‐globin (*HBB*), or gamma‐globin (*HBG1* and *HBG2*), collectively known as HbM disease, which results in the anomaly release of oxygen to the tissues. The final effect is the shifts of the oxygen‐dissociation curve of Hb to the left (right panel). This shift leads to increased affinity of the ferrous iron for oxygen and thus impaired oxygen release to the tissue, resulting in hypoxia with the so‐called functional anemia without Hb decrease.

Given the rarity of these disorders the diagnosis is often delayed, and treatment, particularly, in neonatal/perinatal period may be challenging. Moreover, the management of adult patients in emergency situations with unexpected finding of methemoglobinemia may be dramatic possibly resulting in a delay of therapeutic intervention.

Up today, no guidelines or recommendations on treatment and diagnosis of this conditions exist.

The joined EHA and EuroBloodNet recommendations recently published in the *American Journal of Hematology* (Iolascon A, et al AJH, 2021) included a 22 experts panel, selected for their recognized expertise in research and clinical practice in methemoglobinemia, with a wide geographical representation in order to provide an international perspective (Table 2).^4^ A systematic literature search was performed including the following key search terms “methemoglobin,” “methemoglobinemia,” “inherited methemoglobinemia,” “acquired methemoglobinemia,” “NADH deficiency,” and “*CYB5R3*,” and 92 studies were selected for the panel discussion. Following a Delphi‐like approach, multiple rounds of questionnaires were shared among the panel expert members, a series of questions regarding methemoglobinemia were formulated and discussed up to reach a consensus and produce recommendations.

**Table 2:** Recommendations for Diagnosis and Treatment of Methemoglobinemia

N.	Synthesis of the Recommendations
R.1	It is particularly important to pay attention to clinical findings and family history to help distinguish acquired from inherited forms (whenever these latter are transmitted)
R.2	Key symptoms of methemoglobinemia are related to the MetHb levels. For inherited conditions, these levels range between 10% and 30% that accounts for the occurrence of cyanosis and dark brown blood as main signs. At these levels of MetHb, patients are generally asymptomatic or may present with headaches, tachycardia, and mild dyspnea
R.3	Cytochrome b5 reductase activity measurement is the gold standard test to discriminate hereditary CYB5R3 deficiency from acquired methemoglobinemia. Molecular testing can be considered the gold standard for the diagnosis of hereditary methemoglobinemia
R.4	Management of methemoglobinemia in infancy and childhood is based on the symptoms shown by the patient, the level of MetHb, the cause of the methemoglobinemia, and the patient's age. There are several therapeutic options, the most used are Methylene Blue and Ascorbic Acid. In patients who have developed polycythemia, phlebotomy is not recommended as higher erythrocyte mass allows provision of normal tissue oxygenation
R.5a	Precipitating factors in patients with known hereditary or acquired methemoglobinemia and in patients with a CYB5R3 heterozygous variant should be avoided. It is important to test first degree relatives of patients with hereditary methemoglobinemia
R.5b	In minimally symptomatic or asymptomatic patients, we recommend monitoring without further treatment or addition of oxygen supplementation if needed. In case oxygen is started, monitoring of oxygen saturation with pulse oximetry is usually routinely necessary, at least in neonates. All symptomatic patients should have venous blood MetHb level tested and those without known history of methemoglobinemia should be tested for G6PD deficiency. The first line treatment of the symptomatic patient is MB with a starting dose of 1–2 mg/kg of 1% MB to be repeated up to a dose of 5.5 mg/kg if no response after 30 min. Ascorbic acid can be added as an adjunctive therapy. Patients who do not respond to first line therapy should undergo exchange transfusion or hyperbaric oxygen therapy
R.5c	If MB is to be given to a pregnant patient, the decision should be multidisciplinary and discussed with the patient weighing the risk of hypoxia on the baby and the teratogenic and other effects of MB
R.5d	Potential precipitating and exacerbating factors should be identified prior to surgery. Prophylactic use of MD is recommended only in selected cases of high‐risk patients, like high presurgery MetHb levels or medical history of severe episodes. MB should be prepared and available in the operation room. All patients should receive supplemental oxygen before anesthetic administration. Electrocardiogram monitoring to detect myocardial ischemia and co‐oximeters to identify the MetHb level can be used. Any metabolic abnormality should be corrected before administration of anesthetics. The patient should be monitored during and after surgery for any signs and symptoms of hypoxia
R.6a	Early clinical recognition of methemoglobinemia is of paramount importance. Patients and clinicians should be aware of neurologic and cardiac symptoms and their progression with increasing MetHb values. Prompt referral to specialized laboratories in case of mild symptoms or directly to emergency units in the case of more severe symptoms is fundamental to establish MetHb levels and to start treatment
R.6b	It is recommended to avoid drugs, and chemical substances (possibly present in food, drinks and well water), as well as to promptly treat associated conditions (particularly infections) that may increase methemoglobin levels. A medical alert system is recommended to patients with inherited methemoglobinemia

MB = methylene blue.

In writing the recommendations, the attention was focused on clinical presentation and therapeutic approaches in different periods of life. High percentage of consensus among participants (>90%) has been reached regarding symptoms and diagnostic process.

The diagnosis of methemoglobinemia should be suspected in the case of unexplained cyanosis and hypoxemia; however, the clinical presentation is variable from mildly symptomatic to severe cases.^5^ Cytochrome b5 reductase activity measurement is the gold standard test to discriminate hereditary CYB5R3 deficiency from acquired methemoglobinemia; however, molecular testing, now more and more available since the genes involved are usually included in targeted NGS panels, needs to be performed to confirm the diagnosis and enables to identify congenital forms due to Hb variants^6^, ^7^ (90.9% of consensus on this recommendation). In addition, during the diagnostic process, it is particularly important to pay attention to clinical findings and family history to help distinguish acquired from inherited forms (100% of consensus).

Most of the key symptoms of methemoglobinemia are related to the MetHb levels, that for inherited conditions range between 10% and 30% and accounts for the occurrence of cyanosis and dark brown blood as main signs; at these levels of MetHb, patients are generally asymptomatic or may present with headaches, tachycardia, and mild dyspnea (95.5%).

Again, a very high agreement was reached for clinical management of methemoglobinemia in the neonatal/childhood period: several factors need to be always considered, including whether the patient is symptomatic, the total amount of methemoglobin, the cause of the methemoglobinemia and the patient's age. Symptomatic patients and those with additional factors compromising oxygen delivery (such as congenital heart disease, lung disease, significant anemia, or carbon monoxide poisoning) should be treated at levels between 10% and 30%. In hereditary methemoglobinemia, higher levels of MetHb are better tolerated, with some patients asymptomatic even up to 30%–40%. Infants are at greater risk of developing methemoglobinemia, because of lower levels of erythrocyte CYB5R activity, which is estimated to be around 50%–60% of adult values; in addition, infants have higher levels of HbF which is more readily oxidized to MetHb than adult hemoglobin.^8^, ^9^ Among the several therapeutic options, the most used are Methylene Blue (MB) and Ascorbic Acid (AA).^10^, ^11^ In patients who have developed polycythemia, phlebotomy is not recommended as higher erythrocyte mass allows provision of normal tissue oxygenation. Regarding these aspects, the experts panel reached 86.4% of consensus. Advantages and disadvantages of each approach and dosages defined by patient age have been discussed in completing the recommendations.

In adulthood, the clinical management needs to be adapted at different situations, during daily life, or for example, in emergency situations, in view of surgery, or during pregnancy. The general indication is to try to avoid precipitating factors in patients with known hereditary or acquired methemoglobinemia (also including symptomatic patients where pathogenic CYB5R3 variants have been detected at the heterozygous level, taking always in consideration that genetic testing can fail to identify biallelic variants) (86.4% of consensus).

The direct management of methemoglobinemia during an acute episode requires the stratification of the patients according to the symptoms and MetHb level, and the identification of the precipitating factor. In minimally symptomatic or asymptomatic patients, the experts panel recommends monitoring without further treatment or addition of oxygen supplementation if needed. In case oxygen is started, monitoring of oxygen saturation with pulse oximetry is usually routinely necessary (and particularly indicated in neonates). All symptomatic patients should have venous blood MetHb level tested and those without known history of methemoglobinemia should be tested for G6PD deficiency. The first‐line treatment of the symptomatic patient is 1% MB with a starting dose of 1–2 mg/kg to be repeated up to a dose of 5.5 mg/kg if no response after 30 minutes. AA can be added as an adjunctive therapy. Patients who do not respond to first‐line therapy should undergo exchange transfusion or hyperbaric oxygen therapy (this recommendation reached the 95% of agreement).

Management of methemoglobinemia during pregnancy should be multidisciplinary and always carefully discussed.^12^ Pregnancy is a physiologic state during which there is an increased oxygen demand, and a methemoglobinemia attack can lead to significant morbidity to the fetus due to hypoxia. Moreover, it is well known that MB is teratogenic and should only be used in pregnancy when the risks are felt to outweigh the benefits; therefore, the decision should be discussed with the patient weighing the risk of hypoxia and the teratogenic and other adverse effects on the baby.

Surgery carries a particular risk for patients with known methemoglobinemia because of the well‐established precipitation effect of exposure to anesthetics. Potential exacerbating factors and other comorbidities should be identified before surgery.^13^ Prophylactic use of MB is recommended only in selected cases of high‐risk patients, like high presurgery MetHb levels or medical history of severe episodes.

MB should be prepared and available in the operation room. All patients should receive supplemental oxygen before anesthetic administration. Electrocardiogram monitoring to detect myocardial ischemia and co‐oximeters to identify the MetHb level can be used. Any metabolic abnormality should be corrected before administration of anesthetics. The patient should be monitored during and after surgery for any signs and symptoms of hypoxia (77.3% of consensus).

Finally, the panel expert carefully considered which useful information should be given to patients affected by hereditary methemoglobinemia about their condition, skin color, lifestyle, and drug/food to be avoided. Early clinical recognition of methemoglobinemia is of paramount importance. Patients and clinicians should be aware of neurologic and cardiac symptoms and their progression with increasing MetHb values. Prompt referral to specialized laboratories in case of mild symptoms or directly to Emergency Units in case of more severe symptoms is fundamental to establish MetHb levels and to start treatment.

Several drugs, foods and drinks, and various clinical conditions (particularly infections) may increase the levels of MetHb. It is recommended to avoid such drugs, and chemical substances (possibly present in food, drinks and well water), as well as to promptly treat associated conditions (particularly infections) that may increase methemoglobin levels. A medical alert system is recommended to patients with inherited methemoglobinemia (100% of consensus).

## References

[1] Prchal JT . Methemoglobinemia and other dyshemoglobinemias; Chapter 51. In: Kaushansky K , Prchal JT , Burns LJ et al. , eds. Williams Hematology. 10th ed. New York: McGraw Hill; 2021.

[2] Vichinsky EP , Lubin BH . Unstable hemoglobins, hemoglobins with altered oxygen affinity, and m‐hemoglobins. Pediatr Clin North Am. 1980;27:421–428. 7383714 10.1016/s0031-3955(16)33859-7

[3] Percy MJ , Lappin TR . Recessive congenital methaemoglobinaemia: cytochrome b(5) reductase deficiency. Br J Haematol. 2008;141:298–308. 18318771 10.1111/j.1365-2141.2008.07017.x

[4] Iolascon A , Bianchi P , Andolfo I et al. Recommendations for diagnosis and treatment of methemoglobinemia. Am J Hematol. 2021 September 1. [Epub ahead of print]. doi: 10.1002/ajh.26340 PMC9291883 34467556

[5] Wright RO , Lewander WJ , Woolf AD . Methemoglobinemia: etiology, pharmacology, and clinical management. Ann Emerg Med. 1999;34:646–656. 10533013 10.1016/s0196-0644(99)70167-8

[6] Bianchi P , Vercellati C , Fermo E . How will next generation sequencing (NGS) improve the diagnosis of congenital hemolytic anemia? Ann Transl Med. 2020;8:268. 32355712 10.21037/atm.2020.02.151 PMC7186692

[7] Russo R , Marra R , Rosato BE et al. Genetics and genomics approaches for diagnosis and research into hereditary anemias. Front Physiol. 2020;11:613559. 33414725 10.3389/fphys.2020.613559 PMC7783452

[8] Centorrino R , Shankar‐Aguilera S , Foligno S et al. Life‐threatening extreme methemoglobinemia during standard dose nitric oxide therapy. Neonatology. 2019;116:295–298. 31454813 10.1159/000501462

[9] Greer FR , Shannon M . Infant methemoglobinemia: the role of dietary nitrate in food and water. Pediatrics. 2005;116:784–786. 16140723 10.1542/peds.2005-1497

[10] Zorc JJ , Kanic Z . A cyanotic infant: true blue or otherwise? Pediatr Ann. 2001;30:597–601. 11641851 10.3928/0090-4481-20011001-08

[11] Rino PB , Scolnik D , Fustiñana A et al. Ascorbic acid for the treatment of methemoglobinemia: the experience of a large tertiary care pediatric hospital. Am J Ther. 2014;21:240–243. 24914501 10.1097/MJT.0000000000000028

[12] Grauman Neander N , Loner CA , Rotoli JM . The acute treatment of methemoglobinemia in pregnancy. J Emerg Med. 2018;54:685–689. 29627348 10.1016/j.jemermed.2018.01.038

[13] Kuş A , Berk D , Hoşten T et al. The role of preoperative evaluation for congenital methemoglobinemia. Turk J Anaesthesiol Reanim. 2014;42:223–226. 27366424 10.5152/TJAR.2014.82335 PMC4894152

