# Correction to Summary of Joint European Hematology Association (EHA) and EuroBloodNet recommendations on diagnosis and treatment of methemoglobinemia

**DOI:** 10.1002/hem3.70144

**Published:** 2025-05-03

Iolascon A, Andolfo I, Russo R, et al. Summary of Joint European Hematology Association (EHA) and EuroBloodNet recommendations on diagnosis and treatment of methemoglobinemia. *HemaSphere*. 2021;5:e660. https://doi.org/10.1097/HS9.0000000000000660

The 8th co‐author, incorrectly listed as first name Davis, has been updated. The correct name of this co‐author is David Rees.

The online version of this article has been updated accordingly.

We apologize for this error.

